# Patient education for improving the quality of life among community-dwelling survivors of stroke: A scoping review

**DOI:** 10.51866/rv.694

**Published:** 2026-02-27

**Authors:** Muhammad Arif Razak, Nor Azlin Mohd Nordin, Nor Faridah Ahmad Roslan, Alia A. Alghwiri, Haidzir Manaf

**Affiliations:** 1 Department of Physiotherapy, Hospital Putrajaya, Presint 7, Putrajaya, Wilayah Persekutuan Putrajaya, Malaysia.; 2 Physiotherapy Program, Center for Rehabilitation and Special Needs Studies, Faculty of Health Sciences, Universiti Kebangsaan Malaysia, Kuala Lumpur, Malaysia.; 3 Department of Rehabilitation Medicine, Faculty of Medicine, Universiti Teknologi MARA, Sungai Buloh Campus, Selangor Branch, Jalan Hospital, Sungai Buloh, Selangor, Malaysia.; 4 Department of Physiotherapy, School of Rehabilitation Sciences, The University of Jordan, Amman, Jordan.; 5 Centre for Physiotherapy Studies, Faculty of Health Sciences, Universiti Teknologi MARA, Puncak Alam Campus, Puncak Alam, Selangor, Malaysia.

**Keywords:** Stroke, Cerebrovascular accident, Patient education, Self-care, Quality of life

## Abstract

**Introduction:**

Survivors of stroke frequently encounter difficulties with self-care and self-management, adversely affecting their long-term quality of life (QoL). Patient education is vital in enhancing their QoL, but its overall efficacy must be established. This scoping review aimed to elucidate the efficacy of patient education interventions in improving the QoL of community-dwelling survivors of stroke.

**Methods:**

A scoping review was performed by querying four databases (PubMed, Scopus, Cochrane Library and PEDro) for papers that fulfilled the inclusion criteria. The search terms included (‘stroke’ OR ‘CVA’ OR ‘cerebrovascular accident’) AND (‘patient education’ OR ‘self-care’) AND ‘quality of life’. Publications published in English, subjected to peer review and including human participants were included in the review. Research pertaining to alternative neurological disorders or lacking relevance to the specified subjects of interest was excluded. Two reviewers independently evaluated the full-text papers to determine which ones satisfied the qualifying requirements.

**Results:**

Five studies were included. Patient education interventions demonstrated a consistent trend toward improved quality of life (QoL) among community-dwelling stroke survivors, particularly when programmes were structured, multi-component, and delivered over time. Benefits were observed across domains of functional independence, participation, self-efficacy, and emotional well-being. However, substantial heterogeneity in intervention design and outcome measures limited determination of definitive active components or optimal delivery models.

**Conclusion:**

Patient education interventions hold potential in improving QoL after stroke, but effectiveness is highly dependent on programme design, duration and delivery. Comprehensive, personalised and longitudinally supported interventions are more effective than brief or one-off programmes. Future research should identify core active components, assess subgroup effects and develop adaptable models that can be integrated into routine community-based stroke rehabilitation.

## Introduction

Survivors of stroke frequently encounter a deterioration in physical capabilities, encompassing muscular weakness, impaired coordination and challenges with balance and mobility.^1^ Moreover, they may encounter cognitive deficits, including challenges with memory, attention and problemsolving. Survivors of stroke may also experience melancholy, anxiety and alterations in mood and behaviour. The deterioration in physical, cognitive and emotional functioning can profoundly affect their capacity to execute everyday tasks, participate in social and recreational activities and sustain their autonomy.^2^

A survivor of stroke refers to an individual who has lived beyond the acute phase of a cerebrovascular accident and continues to manage the long-term consequences of the condition.^3^ Quality of life (QoL) is a broad multidimensional concept encompassing physical health, emotional well-being, level of independence, social relationships and personal beliefs.^4^ It was chosen as the primary outcome for this review because it reflects the overall impact of stroke and rehabilitation on an individual’s ability to live meaningfully and independently in the community.

While this review primarily focuses on survivors of stroke, it is important to acknowledge that educational interventions may indirectly benefit caregivers by enhancing survivors’ selfmanagement abilities and reducing caregiving burden. Improved survivor outcomes, particularly QoL and independence, can alleviate emotional and logistical challenges faced by family members and caregivers. However, this review does not specifically evaluate caregiver outcomes.

Presently, educational programmes for survivors of stroke often encompass information regarding stroke prevention, risk factor management, medication compliance, rehabilitation exercises and community resources. These programmes equip individuals with essential knowledge and skills for efficient recovery management, leading to enhanced self-management abilities and greater understanding of stroke recovery.^5^ These therapies are frequently administered by healthcare professionals, such as nurses, occupational therapists or speech-language pathologists.

Research has consistently demonstrated that patient education programmes result in substantial enhancements in the QoL of community-dwelling survivors of stroke. These enhancements may include heightened life satisfaction, superior emotional well-being and improved social involvement.^6^ Furthermore, patient education programmes have shown the potential to augment physical function, including mobility, strength and coordination.^7^ They also improve self-efficacy by empowering survivors of stroke with the information and skills necessary to take an active role in their rehabilitation and health management.^8,9^

Although several studies indicate beneficial outcomes such as knowledge enhancement and behavioural modification, the overall effectiveness of patient education in improving QoL remains inconclusive.^10,11^ This inconsistency may result from variations in intervention content, delivery methods, population characteristics and outcome measures.^6^ Therefore, further synthesis is necessary to determine which approaches are most effective and under what conditions.

Patient education programmes can be administered through diverse modalities, facilitating flexibility and accessibility. In-person workshops facilitate direct engagement with healthcare specialists, enabling tailored assistance and immediate feedback. Online programmes offer convenience and can be accessed from home, supporting individuals with mobility limitations or transportation challenges. Printed materials, such as brochures and pamphlets, provide tangible resources that survivors of stroke can refer to as needed.^12^ Each method has its own strengths and weaknesses, and the choice of format should depend on the individual’s preferences and needs.

Although patient education programmes for survivors of stroke have demonstrated efficacy, certain limitations warrant attention. A key challenge is accessibility, as not all survivors of stroke may have equal access to in-person or online options. Additionally, people have varying learning styles and preferences, which may influence the effectiveness of programmes.^13^ The knowledge and skills gained may also require ongoing reinforcement to ensure sustained behavioural change and long-term benefits. Addressing these challenges is critical to maximising the impact of educational interventions in stroke rehabilitation.

This scoping review sought to map and assess patient education interventions targeting community- dwelling survivors of stroke, with a specific focus on their effects on QoL outcomes. It aimed to clarify the overall efficacy of these interventions, identify effective characteristics and delivery methods and highlight areas requiring further research.

## Methods

The review protocol has been registered with the Open Science Framework (https://osf.io/ym8sg/). This scoping review followed the methodological framework proposed by Arksey and O’Malley and adhered to the Preferred Reporting Items for Systematic Reviews and Meta-Analyses Extension for Scoping Reviews (PRISMA-ScR).

Electronic searches were conducted in the following four databases to ensure a broad and representative coverage of relevant literature: PubMed, Scopus, Cochrane Library and PEDro. PubMed provides extensive access to biomedical literature; Scopus offers multidisciplinary scientific literature; the Cochrane Library is known for its systematic reviews and clinical trials; and PEDro is specific to physiotherapy and rehabilitation research.

The search strategy involved the use of the following keywords: ‘stroke’, ‘CVA’, ‘cerebrovascular accident’, ‘patient education’, ‘self-care’ and ‘quality of life’. Boolean operators (‘AND’, ‘OR’ and ‘NOT’) were applied to refine the results. The search was conducted from 18 to 20 December 2023 and limited to English-language articles published from 2013 to 2023. The timeframe beginning in 2013 was selected to capture the most recent decade of literature reflecting current trends in stroke education and rehabilitation.

In addition to the database search, the reference lists of relevant studies were reviewed to identify further eligible articles. Despite inclusion of the Cochrane Library, systematic reviews and narrative reviews were excluded from this scoping review to focus exclusively on primary research evidence.

The eligibility criteria were defined using the population, concept and context framework: population: community-dwelling survivors of stroke; concept: patient education interventions; and context: studies reporting on education programme characteristics and QoL outcomes. Additional inclusion criteria required that studies (1) be published in English, (2) describe patient education strategies for community-dwelling survivors of stroke, (3) report on the methodology and characteristics of interventions and (4) assess outcomes related to QoL.

Studies not meeting the abovementioned criteria, including non-primary sources, were excluded. Specific study designs targeted included randomised controlled trials (RCTs), quasi-experimental studies and observational studies reporting original data.

All identified records were imported into a reference management software for duplicate removal. Two reviewers independently screened titles and abstracts against the eligibility criteria. Articles that met the inclusion criteria or whose eligibility was uncertain were retrieved in full and assessed independently. Discrepancies in inclusion decisions were resolved through discussion or, if necessary, consultation with a third reviewer.

Data extraction was conducted independently by two reviewers using a structured Excel form. Extracted data included authorship, year of publication, country, study design, characteristics of interventions (e.g. duration, delivery mode, content, provider and contact hours), outcome measures related to QoL, significance of findings, adherence and any reported adverse events. Two additional reviewers verified the accuracy of the extracted data.

An inductive thematic approach was used to synthesise the data. Key information from the included studies was organised into themes such as delivery formats including in-person, online or printed materials; frequency and duration of interventions; outcomes measured (primarily QoL indicators); and study findings. The themes were derived by identifying patterns across studies and clustering similar intervention features and effects. This process allowed for a comprehensive understanding of patient education interventions in the community-dwelling population with stroke.

The synthesis aimed to map the scope of available literature, summarise key intervention characteristics and highlight gaps for future research. This structured approach enabled an indepth evaluation of the effectiveness and implementation of patient education programmes for community-dwelling survivors of stroke.

## Results

A total of 224 records were identified through comprehensive searches conducted in the four databases: PubMed, Scopus, Cochrane Library and PEDro. No additional records were obtained from other sources. After the removal of 16 duplicate entries, 208 records were screened based on their titles and abstracts. Of them, 176 were excluded for not meeting the predefined inclusion criteria. The majority of these exclusions were due to studies not involving populations with stroke, lacking relevance to patient education or omitting outcomes related to QoL.

**Figure 1:**
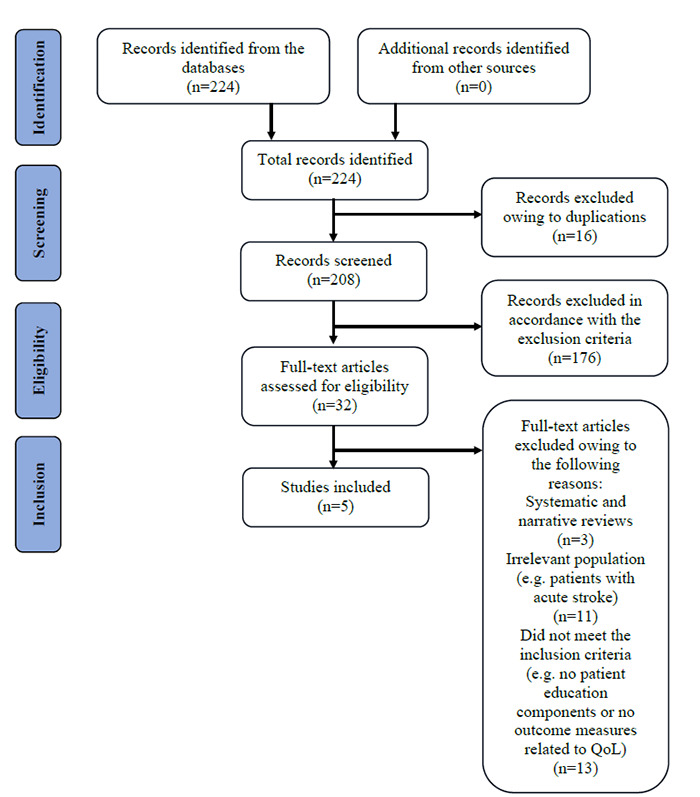
PRISMA-ScR flowchart of the scoping review.

Following the initial screening phase, 32 full-text articles were retrieved and assessed for eligibility. Upon full-text review, 27 articles were excluded for specific reasons. Three articles were identified as systematic or narrative reviews, which were excluded to maintain a focus on primary research. Eleven studies were excluded because they targeted populations not relevant to this review, such as individuals with acute stroke or those in institutional settings. Thirteen articles were removed for failing to report relevant patient education components or outcome measures related to QoL. A total of five studies were included in the final synthesis. The selection process is summarised in Figure 1 (PRISMA-ScR flowchart).

### Study description

Five studies,^5,10,14-16^ published from 2013 to 2022, were included, involving a total of 628 community-dwelling survivors of stroke across Australia, Italy, the United Kingdom, Taiwan and Colombia. Three^5,10,14^ were RCTs, and two15,16 were feasibility studies. Their sample sizes varied widely, with those of intervention groups ranging from 10 to 328 participants and control groups from 10 to 300.

The participants were typically middle-to-older-aged adults (mean age: 55–76 years). Across studies,^5,10,14-16^ survivors of stroke were operationally defined as individuals with confirmed ischaemic or haemorrhagic stroke who were medically stable and residing in the community. Most studie^s5,10,14-16^ excluded those with severe cognitive impairment, although those with mild- to-moderate deficits were eligible. Independence in activities of daily living (ADL) was generally required at baseline to ensure that participants were able to engage with the interventions.

### Intervention characteristics

The interventions demonstrated considerable heterogeneity in design, delivery and duration, ranging from short 1-to-2-week digital interventions to 6-month or even multiyear programmes. Delivery formats included group-based physical activity combined with education,^10,14^ individualised rehabilitation sessions embedding self-management education,^16^ mobile application-based education^15^ and telehealth lifestyle coaching.^5^ Content also varied, with exercise-plus-education models focusing on physical activity, adherence and lifestyle modification; self-management programmes emphasising autonomy, problem-solving and goal-setting; and digital interventions (mobile application and telehealth) targeting risk factor awareness, lifestyle habits and ongoing support. All interventions were delivered by trained healthcare professionals, including physiotherapists, occupational therapists and stroke specialists, ensuring high-quality educational input, while control groups typically received usual care such as routine rehabilitation, clinical follow-up or written information without structured patient education programmes.

### Outcome measures

QoL was not the primary outcome in most studies.^5,10,14-16^ Several validated instruments were used to measure QoL and assess the effectiveness of the interventions. The most frequently utilised tools were the Short-Form 12 (SF-12) and Short-Form 36 (SF-36) Health Surveys^5,10,16^ and the EuroQoL 5-Dimension (EQ-5D-3L/5L).^14,15^ Additionally, some studies employed condition- specific tools such as the Stroke and Aphasia Quality of Life (SAQOL).^16^

### Adverse events and adherence

Adherence was generally high across the included studies and referred not only to safety but also to session attendance, engagement with educational content and compliance with prescribed exercise or self-care activities. Most interventions were well tolerated, with adverse events infrequently reported and primarily associated with the physical activity components rather than the educational elements. Brauer et al.^14^ reported attendance rates above 80% and no adverse effects during treadmill training combined with education. Calugi et al.^10^ documented a high dropout rate due to musculoskeletal pain and fatigue during exercise sessions, although those who remained in the programme demonstrated strong adherence. Jones et al.16 noted good participation but did not provide details on adverse events, limiting conclusions about intervention safety. Kang et al.^15^ observed high levels of application engagement, particularly among younger participants, and reported no adverse events, although underreporting could not be excluded. Sakakibara et al.^5^ achieved adherence rates exceeding 85% with telehealth lifestyle coaching and comprehensively monitored participant safety, documenting two deaths during follow-up that were unrelated to the intervention. Taken together, the evidence suggests that patient education interventions are generally safe, with minimal risk attributable to the educational components themselves, and adherence levels are consistently strong across delivery modes.

### QoL

QoL outcomes were assessed using a range of validated instruments, including the EQ-5D, SF-12, SF-36 and SAQOL, with results demonstrating considerable variability depending on intervention type and duration. Short-term or informational interventions, such as the application-based programme by Kang et al.^15^ and the Bridges self-management programme reported by Jones et al.,^16^ showed no significant improvements in QoL scores, highlighting the limited impact of brief or minimally interactive education. Exercise-plus-education interventions produced more mixed results: Brauer et al.^14^ reported stable EQ-5D scores with no significant change in self-perceived health despite improved adherence, while Calugi et al.^10^ observed fluctuations in both physical and mental components of the SF-12. Although the overall effect in the trial by Calugi et al. was not statistically significant (P>0.05), their subgroup analysis suggested that participants with higher baseline physical function experienced greater benefit, pointing to potential differential responsiveness. By contrast, Sakakibara et al.^5^ reported the most robust outcomes, with telehealth lifestyle coaching producing significant improvements in physical health-related QoL (SF-36 PCS, P<0.05), sustained at both 6- and 12-month follow- ups. These findings collectively indicate that while short or one-off educational strategies are unlikely to yield meaningful changes in QoL, multicomponent, longitudinal interventions that combine education with ongoing coaching and behavioural support have greater potential to produce lasting improvements in the well-being of community-dwelling survivors of stroke.

**Table 1:** Characteristics of the included studies.

Author (year)	Country	Patient	Type of Intervention and Control						Intervention	Outcome measure (QOL)	Significance of the outcome measure of QOL
			Period	Mode	Delivery	Content	Contact hour	Provider			
Brauer et al. (2022)^14^	Australia	IG: 60 CG: 59	**IG:** Gait training using high-intensity treadmill training plus self-management **CG:** Usual gait training	26 weeks	Group	F2F	Receive a workbook at the study’s outset. The workbook is used for selfmonitoring and goal-setting. Behavioural change techniques include education, selfmonitoring, goalsetting, feedback and problem-solving. Risk perception and outcome expectancies are addressed. Task self-efficacy is enhanced through instruction and feedback. Short-, mediumand long-term goals are set. Action planning and coping planning are conducted.	30 minutes, three times a week for 8 weeks; the other part of the week involves usual rehabilitation	Physiotherapists with more than 5 years of clinical experience or experience in treadmill training or selfmanagement	EQ-5D Weeks 8 and 26	EQ-5D: ↔
Calugi et al. (2016)^10^	Italy	IG: 126 CG: 103	**IG:** APA, enhanced with TPE (APA-TPE) **CG:** Usual care	4 months	Group	F2F	First session: causes of stroke, modifiable and nonmodifiable risk factors, impairment, disabilities and limitations in participation, course of illness Second session: disabilities related to stroke, use of aids, how to adapt the home environment to one’s needs Third session: benefits of physical exercise to maintain a healthy lifestyle	One-hour physical exercise programme twice a week over an 8-week period (16 sessions)	One session was held by a physician, and two sessions were held by physical therapists	SF-12	SF-12: Physical composite summary: ↑↓ Mental composite summary ↑↓
Jones et al. (2016)^16^	United Kingdom	Clusters randomised: IG: site 1: 16 IG site 2: 24 CG site 1: 22 CG site 2: 16	**IG:** Introduced to the stroke workbook and the seven key principles of selfmanagement by the therapist integrated into existing CSR sessions **CG:** Received CSR as usual, which included access to physiotherapy, occupational therapy and speech and language therapy, if required	12 weeks	Individual	F2F	The Bridges self-management programme aimed to be distinct from routine stroke rehabilitation provision in two main ways: 1. One-to-one rehabilitation sessions using seven principles integrated into each therapy session to support self-management activities 2. A stroke workbook that included vignettes, activities, ideas and solutions from other survivors of stroke for successful selfmanagement and space to record and reflect goals and progress.	Baseline, 6 weeks and 12 weeks	Occupational therapists, physiotherapists, speech and language therapists and rehabilitation support workers	SF-12 and SAQOL	SF-12 ↔ SAQOL ↔
Kang et al. (2019)^15^	Taiwan	IG: 38 CG: 38	**IG:** SHEMA **CG:** Stroke health education booklet.	30 days	Individual	F2F for booklet and mobile application explanation Educational technology for IG	12 topics of risk factors in patients with stroke such as stroke history, heart disease, age, irregular work and sleep patterns, obesity, family history and genetic factors, hyperlipidaemia, hypertension, unbalanced diet, diabetes mellitus, changes in ambient temperature and sex	Duration of booklet and mobile application explanation: approximately 45 minutes Patients read the booklet or SHEMA content at home for 7–14 days, with a minimum requirement of 5 minutes per day	Research assistant	EQ-5D and EQ-5D VAS	EQ-5D ↔ EQ-5D VAS ↔
Salazar et al. (2017)^5^	Colombia	IG: 64 CG: 62	**IG:** Memory training programme. **CG:** Usual care.	12 months	Individual	Phone calls	**Stroke Coach:** Lifestyle coaching to facilitate motivation for behavioural change Stroke selfmanagement manual Self-monitoring kit: Health report card Blood pressure monitor Pedometer Food/physical activity diary Tape measure and Body Mass Index (BMI) guidelines Instructions Memory training programme: Coaches educated participants about memory and memory after stroke Subjects learnt about internal and external strategies to enhance memory as well as the influence of beliefs, anxiety, worry and motivation on memory.	Seven telephone sessions (30–45 minutes), five follow-up calls (5–10 minutes) (6 months)	All coaches were health professionals or research assistants with university training experienced in working with individuals with stroke	SR-36	SF-36 ↑ ↓

Arrows indicate significant (↑↓) or non-significant (↔) differences between the interventions and controls unless otherwise stated. APA: adaptive physical activity, CG: control group, CSR: community stroke rehabilitation, EQ-5D-3L: EuroQoL 5-Dimensional Scale, F2F: face-to-face, IG: intervention group, SAQOL: Stroke and Aphasia Quality of Life, SF-12: Short-Form 12 Health Survey, SF-36: Short-Form 36 Health Survey, SHEMA: stroke health-education mobile application, TPE: therapeutic patient education

**Table 2:** Summary of the intervention characteristics in the included studies.

Author (year and country)	Study design	What? (Intervention)	Who? (Provider)	How? (Mode and format)	When? (Duration and frequency)	Where? (Setting/platform)	CG
Brauer et al. (2022)^14^ Australia	RCT	Treadmill training and structured self-management education (goal-setting, problem-solving, lifestyle adaptation)	Physiotherapists and stroke specialists	Group sessions, supervised	30 minutes, three times per week, 8 weeks and routine rehabilitation	Hospital rehabilitation centre	Usual care (routine rehabilitation only)
Calugi et al. (2016)^10^ Italy	RCT	Adapted physical activity plus TPE (prevention, adherence, psychosocial adjustment)	Physiotherapists	Group sessions, interactive	1 hour, two times per week, 8 weeks (16 sessions total)	Rehabilitation centre	Standard rehabilitation (no TPE)
Jones et al. (2016)^16^ United Kingdom	Feasibility study	Bridges self-management programme (individual coaching, workbook- based reflection, autonomy- focused)	Trained rehabilitation staff	One-on-one sessions and workbook	Three sessions (baseline, week 6, week 12); duration not specified	Outpatient/community rehabilitation	Usual care rehabilitation
Kang et al. (2019)^15^ Taiwan	Feasibility study	Mobile application-based stroke education plus booklet (risk factor awareness, lifestyle modification, self-care strategies)	Research staff and health educators	Digital (introductory session and daily application use)	45-minute introductory session and daily use at home (1-2 weeks)	Home-based, mobile application platform	Standard post-stroke outpatient education
Sakakibara et al. (2022)^5^ Colombia	RCT	Telehealth lifestyle coaching (exercise, diet, stress management, medication adherence)	Stroke coaches and clinicians	Telehealth (video calls and follow-up phone check-ins)	30-45 minutes per session, over 6 months	Remote (telehealth platform)	Usual care (no structured coaching)

CG: control group, RCT: randomised controlled trial, TPE: therapeutic patient education

**Table 3:** Summary of the outcome measures related to QoL.

Author (year and country)	QoL instrument	Reported outcome	Statistical finding	Note/subgroup effect
Brauer et al. (2022)^14^ Australia	EQ-5D	No significant change in QoL scores post-intervention	P>0.05 (not significant)	Despite high adherence and improved physical activity, the perceived health status did not change.
Calugi et al. (2016)^10^ Italy	SF-12	Mixed changes: Some participants improved, while others declined.	No overall significant group effect (P>0.05)	Participants with higher baseline physical function benefitted more → possible subgroup responsiveness.
Jones et al. (2016)^16^ United Kingdom	SF-12 and SAQOL	No consistent improvements in physical or emotional QoL domains	No P-values reported	Feasibility trial; limited sample and frequency of sessions may explain the lack of effect.
Kang et al. (2019)^15^ Taiwan	EQ-5D and EQ-5D VAS	No significant changes in QoL post-application use	P>0.05	Short intervention period (1–2 weeks) likely insufficient to affect QoL outcomes
Sakakibara et al. (2022)^5^ Colombia	SF-36	Significant improvements in physical component scores sustained at 6 and 12 months	P<0.05; CI not fully reported	Longitudinal telehealth coaching provided lasting improvements in physical health-related QoL. Mental health improvements were less consistent.

## Discussion

This review highlights both the promise and limitations of patient education in post-stroke rehabilitation. While education is widely regarded as essential, its effectiveness in improving QoL is context-dependent, influenced by intervention design, duration and delivery format as well as participant characteristics.

Programmes of limited intensity or duration, such as application-based education or infrequent self-management sessions, were generally ineffective in producing QoL gains. Exercise-plus- education approaches increased adherence and participation but did not consistently affect selfreported well-being, with some evidence of subgroup-specific benefit. In contrast, the telehealth lifestyle coaching programme demonstrated significant and sustained improvements in physical health-related QoL. Its success likely stemmed from its intensity, comprehensive scope, personalised content and longitudinal reinforcement, which together promoted meaningful behavioural change.

For clinical practice, the findings suggest that patient education is most effective when integrated into multicomponent, patient-centred and sustained rehabilitation frameworks. Key features such as goal-setting, problem-solving, behavioural coaching and follow-up support are essential to translating education into long-term improvements. Digital tools offer promising avenues for accessibility and scalability but require integration with ongoing coaching and consideration of participants’ digital literacy and socio-cultural context.

Future research should aim to identify the active components of effective education, assess subgroup responsiveness and incorporate economic and implementation evaluations to inform scalability. Longitudinal RCTs with robust follow-up are needed to confirm the sustainability of effects, while research in low-resource settings will enhance generalisability.

### Limitations

This review is subject to several limitations. Only English-language publications were included, potentially excluding relevant studies published in other languages. The search strategy was confined to four databases, which, while comprehensive, may not have captured all eligible studies. Furthermore, as a scoping review, this study did not include a formal appraisal of methodological quality, limiting the ability to assess the strength of evidence.

Several included studies had small or unrepresentative samples, varied designs (e.g. non-randomised trials) and short follow-up periods. Additionally, intervention fidelity, digital access and participant adherence varied and were inconsistently reported. These limitations must be considered when interpreting the findings and drawing implications for practice.

## Conclusion

Patient education interventions hold promise in improving QoL among community-dwelling survivors of stroke, but their effectiveness depends on design and delivery. Short, informational approaches are insufficient, whereas comprehensive, personalised and longitudinally supported programmes such as telehealth lifestyle coaching show the strongest potential for sustained benefits. Future efforts should focus on defining the active components of effective education and developing adaptable but standardised models that can be integrated into routine community-based stroke rehabilitation.

## Review protocol registration

The review protocol has been registered with the Open Science Framework (https://osf.io/ym8sg/).
